# Enhancing Kidney Transplant Outcomes: The Impact of Living Donor Programs

**DOI:** 10.3390/jpm14040408

**Published:** 2024-04-12

**Authors:** Andrea Noya-Mourullo, Alejandro Martín-Parada, Alberto Palacios-Hernández, Pablo Eguiluz-Lumbreras, Óscar Heredero-Zorzo, Francisco García-Gómez, José Luis Álvarez-Ossorio-Fernández, Andrea Álvarez-Ossorio-Rodal, Magaly-Teresa Márquez-Sánchez, Javier Flores-Fraile, Pilar Fraile-Gómez, Bárbara Yolanda Padilla-Fernández, María-Fernanda Lorenzo-Gómez

**Affiliations:** 1 Urology Department, University Hospital of Salamanca, 37007 Salamanca, Spain; id00639980@usal.es (A.N.-M.); oscarheredero@usal.es (Ó.H.-Z.); fgarciagomez1@saludcastillayleon.es (F.G.-G.);; 2 Department of Surgery, University of Salamanca, 37007 Salamanca, Spainmagalymarquez@usal.es (M.-T.M.-S.); 3 Urology Department, Puerta del Mar University Hospital in Cádiz, 11009 Cádiz, Spain; 4 Nephrology Department, University Hospital of Salamanca, 37007 Salamanca, Spain; mpfraile@saludcastillayleon.es; 5 Urology Unit, Surgery Department, University of La Laguna, San Cristóbal de La Laguna, 38200 Santa Cruz of Tenerife, Spain

**Keywords:** transplant, living donor programs, kidney transplant, kidney donor

## Abstract

Introduction: The protocol for deceased donor kidney transplants has been standardised. The procedure for a living donor has peculiarities derived from the differences in the graft. When a living kidney donor program is implemented, changes occur in both the profile of the kidney transplant candidate and in the postoperative treatments. Aims: To discover whether a living donor program influences the functional outcomes of kidney grafts in a longstanding classical deceased donor kidney transplant program and to identify the factors associated with transplant outcomes. Methods: Retrospective observational multicentre study. Sample: Kidney transplant patients in two urology referral centres for renal transplant in Spain between 1994 and 2019. Groups: TV (living transplant): patients given kidney transplants from living donors (*n* = 150); TCpre11 (deceased transplant previous to 2011): patients given kidney transplants from deceased donors before the living donor program was implemented (*n* = 650); and TCpost11 (deceased transplant after 2011): patients given kidney transplants from deceased donors after the living donor program was implemented (*n* = 500). Results: Mean age was 55.75 years (18–80 years), higher in TCpre11. There were 493 female patients (37.92%) and 1007 male patients (62.08%). Mean body mass index (BMI) was 26.69 kg/m^2^ (17.50–42.78 kg/m^2^), higher in TCpre11. Mean ischemia time was 17.97 h (6–29 h), higher in TCpost11. Median duration of urethral catheter: 8 days (6–98 days), higher in TCpost11. Median duration of double-J ureteral stent: 58 days (24–180 days), higher in TCpost11. Pretransplant UTIs: 17.77%, higher in TCpre11 (25.69%) than in TV (12%), higher in TV (12%) than TCpost11 (9.2%), and higher in TCpre11 (25.69%) than TCpost11 (9.2%). Acute renal rejection in 9.33% of TV, 14.77% of TCpre11, and 9.8% of TCpost11. Multivariate analysis: TCpost11 featured higher BMI, more smoking, and chronic renal failure progression time. Lower use of nonantibiotic prophylaxis to prevent recurrent urinary tract infections, increased duration of urethral catheters due to obstructive problems, and favoured deterioration of kidney function was observed in the deceased donor program. The living donor (LD) program had a strong influence on deceased donor transplants in the prelysis phase. Implementation of a LD program was associated with a decrease in the likelihood of acute rejection in TCpost11 and an increase in the tendency towards normal kidney function. Conclusions: Implementing living donor transplant programs affects functional outcomes in deceased donor transplants, reducing the probability of acute rejection and increasing the tendency towards normal kidney function. Preventing recurrent urinary tract infections with measures other than antibiotics, smoking cessation, delaying the removal of the double-J stent from the graft, and pre-emptive transplant (transplant prior to dialysis) are associated with improved renal function of the graft.

## 1. Introduction

Global prevalence of chronic kidney disease (CKD) is on the rise due to increased prevalence of hypertension (HT), diabetes mellitus (DM), and cardiovascular disease (CVD), as well as the increasing longevity of the population. The prevalence of CKD is estimated at 12.7%, rising to 21.4% in people over 64 [1].

Kidney transplants are currently the treatment of choice for patients with CKD [2], producing more favourable outcomes in terms of patient morbidity, mortality, and quality of life with respect to renal replacement therapy, in addition to a clear improvement in overall survival rates [2]. Spain is the world leader in number of kidney transplants, with a total of 3688 transplants performed in 2023 [3]. In spite of these numbers, in recent years, one problem, the existence of waiting lists, has become noticeable across all public health sectors, affecting an ageing [4] and increasingly fragile [5] population.

Living donor kidney transplantation has emerged as an alternative treatment to renal replacement therapy in societies with more restrictions on deceased organ donation. In Spain, a total of 442 living donor transplants were performed in 2023, contrasting with the 3246 deceased donor transplants registered by the National Transplant Organisation (Organización Nacional de Transplantes, ONT) [3]. 

The surgical technique for deceased donor transplants has been standardised and is described in manuals for both general and urological surgery [6]. 

Living donor surgery has peculiarities derived from the differences in the graft, which is extracted from a healthy person [7].

Living and deceased donor transplants in Spain are regulated by the Royal Decree 1723/2012, 28 December 2012, in which the following requirements are stated: Donors must be of age, have full mental faculties and good health, not suffer from any mental illness that could prevent consent, and be informed of the risks and consequences of organ donation. In addition, the process includes a medical and psychiatric evaluation of the donor by different physicians than those who will perform the transplant, and the donor must appear before a judge to express their desire to donate an organ and give informed consent to it.

A deceased donor is a deceased person from whom it is intended to obtain organs for their subsequent transplant and that, by the requirements established in the law, would not have expressly stated their opposition. In this type of donation, the diagnosis of death is the confirmed and irreversible cessation of circulatory and respiratory functions or brain functions, thus including both encephalic death and circulatory and respiratory death thanks to the preservation techniques that can be used in the latter [8].

The applications and regulations that this law enforces are meant to provide a secure way to control organ donations and protection for the person who decides to donate and the potential donor. It also works as a guide to all centres that perform organ donation procedures, facilitating order in the process and promoting organ donation. 

As published in the memoir by the University Hospital of Salamanca about donation and transplant coordination to promote living donor programs in the region, the hospital’s coordination of transplants and transplant team certification, according to the ISO Quality Management system 9001/2015, has been audited and given recertification until 2025; the University Hospital of Salamanca also participates in teaching activities regarding organ transplant for incoming personal and other regions of Castilla y Leon. The hospital’s transplant coordination personnel also need to participate in training activities about transmissible diseases, surgical techniques, individualising basis, and intensive and critical medicine [9]. 

In addition to academic activities, the hospital’s transplant coordination personnel participate in a wide range of activities with the region’s population like teaching about this topic in schools, participating in award ceremonies, public races to promote visualisation of kidney patients and recruitment of donors, distribution of photography related to donation and transplant, including preparation of annual calendars with these photos, and participation in social networks, either their own or through associations, for data dissemination and public awareness about the subject [9].

Since a living kidney donor program was implemented at our healthcare centre in 2011, there have been changes in both the profile of candidate kidney transplant patients, due to epidemiological changes found in an ageing population, and in the treatments employed during the early and late postoperative period.

The aims of the present study are to ascertain whether implementing a living donor program influences functional outcomes of kidney grafts in a longstanding, classical deceased donor kidney transplant program, to identify factors related to renal function in a deceased donor program in the period following the implementation of a living donor program, and to compare these with factors prior to the implementation of living donor transplants.

## 2. Methods

A retrospective observational multicentre study was conducted.

The sample consisted of kidney transplant patients in the Urology Service of the Salamanca University Hospital (37007 Salamanca, Spain) and in the Urology Service of Puerta del Mar University Hospital in Cádiz (11009 Cádiz, Spain) between 19 October 1994 and 18 September 2019. Inclusion criteria: patients over 18 years of age, diagnosed with end-stage kidney disease, and given kidney transplants from a deceased or living donor. Exclusion criteria: patients under 18 years of age and kidney transplant recipients who did not sign an informed consent form before surgery authorising use of data for scientific purposes.

Groups:

**TV**: patients given kidney transplants from living donors (*n* = 150).

**TCpre11**: patients given kidney transplants from deceased donors before the living donor program was implemented (*n* = 650).

**TCpost11**: patients given kidney transplants from deceased donors after the living donor program was implemented (*n* = 500).

Variables: age, body mass index (BMI), urine cultures, pre- and post-transplant haemoglobin level, pre- and post-transplant creatinine level, pre- and post-transplant blood pressure, ischemia time, days of urethral catheter, days of double-J stent, sex, pretransplant urinary tract infections, treatment of pretransplant urinary tract infections, post-transplant Doppler ultrasound results, reason for transplant, immunosuppressive treatment, concomitant diseases, surgical history, concomitant treatments, results of additional tests, dialysis treatment, post-transplant dialysis, acute graft rejection, and functional outcomes. Four functional outcomes were distinguished: (a.) normal kidney function; (b.) graft complication without loss of kidney function; (c.) graft complication involving loss of kidney function; and (d.) graft loss.

Results were analysed with descriptive statistics, Student’s *t*-test, Chi2, Fisher’s exact test, ANOVA analysis of variance (Scheffe’s test for normal samples and Kruskal–Wallis for other distributions), Pearson and Spearman correlation, cluster analysis, and multiple regression. The software used to implement the analyses was SPSS V25 automatic statistical package, IBM Corp. Released 2017 and IBM SPSS Statistics for Windows, Version 25.0. Armonk, NY, USA: IBM Corp. *p* < 0.05 was accepted as significant.

The research protocol PI 2023011200 was approved by the Ethics Committee for Clinical Research of the University Hospital of Salamanca. The authors declare that there are no conflicts of interest.

## 3. Results

**Figure 1:**
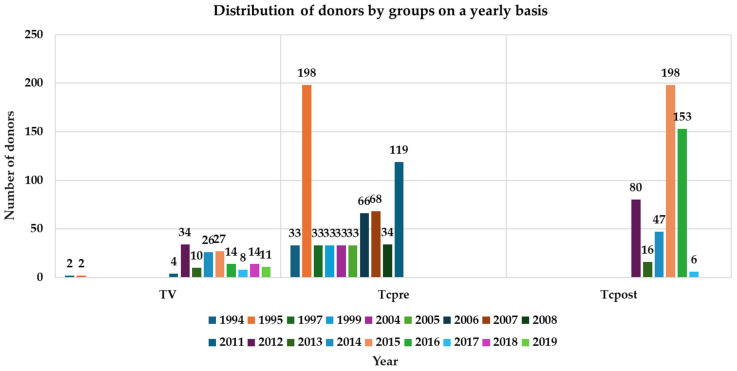
Distribution of donors by groups on a yearly basis.

The distribution of the donors in each group, and by year, is reported in Figure 1.

The living donors were categorized into two types: family related (75.33%) and crossover (24.66%, *p* = 0.0001), in the family category the most frequent was sibling (56%), parent (16.66%), spouse (2%), and children (0.66) *p* = 0.0001.

Mean age was 55.75 years (SD 15.38), with a range of 18–80 years, and was higher in TCpre11 (*p* = 0.0004). A total of 37.92% of patients were female and 62.08% were male. Mean BMI was 26.69 kg/m^2^, with a SD of 4.74, a median of 25.91, a range of 17.50–42.78, and was higher in TCpre11 (*p* = 0.0002). Mean count of positive urine cultures was 3.04, with a SD of 3.16, a median of 2.00, and a range of 0–13, with the highest number of positive cultures occurring in TCpre11 (*p* = 0.0047). In TCpre11, the mean creatinine level was 2.73 mg/dL, with a SD of 3.68, a median of 1.60, and a range of 0.60–61.60 and was higher in TCpre11 (*p* = 0.007). Mean systolic blood pressure was 135.43 mm/Hg, with a SD of 18.86, a median of 134.00, a range of 73–190, and was higher in TCpost11 (*p* = 0.0001). Mean diastolic blood pressure was 76.34 mm/Hg, with a SD of 12.46, a median of 75.00, a range of 48–111, and was higher in TCpost11 (*p* = 0.0074). In TCpost11, the mean haemoglobin level was 12.10 g/dL, with a SD of 1.30, a median of 12.25, and a range of 11.00–15.80, with no differences between groups (*p* = 0.056). Mean creatinine level was 1.72 mg/dL, with a SD of 1.14, a median of 1.40, and a range of 0.59–10.40, with a higher creatinine level in TCpost11 (*p* = 0.0026). Mean systolic blood pressure was 125.95 mm/Hg, with a SD of 9.74, a median of 130.00, a range of 100–145, and was higher in TCpost11 (*p* = 0.0001). Mean diastolic blood pressure was 75.71 mm/Hg, with a SD of 9.95, a median of 75.00, a range of 60–97, and was higher in TCpost11 (*p* = 0.0002) (Supplementary Materials: Table S1).

**Figure 2:**
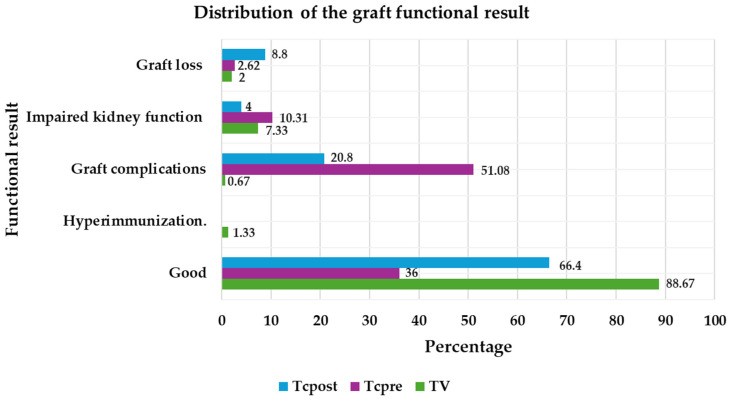
Distribution of the graft function results.

The cause of more frequent graft failure was impaired kidney function in the TV group (7.33%); Graft complications that do not involve loss, including lithiasis, glomerulopathy, drug toxicity, cysts, virus infection, ectasia, ureteral reimplantation, reflux, and urinary tract infections, were more frequent in groups TCpre (51.08%) and TCpost (20.80%). Good functional results were more frequent in the TV group (88.675%) (Figure 2).

Mean ischemia time was 17.97 h, with a SD of 5.39, a median of 18.00, a range of 6–29, and was higher in TCpost11 (*p* = 0.0001). Mean duration of urethral catheterization was 12.64 days, with a SD 15.22, a median of 8.00, a range of 6–98, and with the longest duration occurring in TCpost11 (*p* = 0.0003). Mean duration of double-J stent was 56.11 days, with a SD of 18.85, a median of 58.00, a range of 24–180, and was higher in TCpost11 (*p* = 0.0006). Pretransplant UTIs occurred in 17.77% of patients, and the rate was higher in TCpre11 (25.69%) than TV (12%), with a *p*-value = 0.0002; was higher in TV (12%) than TCpost11 (9.2%), with a *p*-value = 0.3481; and was higher in TCpre11 (25.69%) than in TCpost11 (9.2%), with a *p*-value = 0.0001. The distribution of acute renal rejection in the groups was 9.33% in TV, 14.77% in TCpre11, and 9.8% in TCpost11. (Supplementary Materials: Table S2).

**Figure 3:**
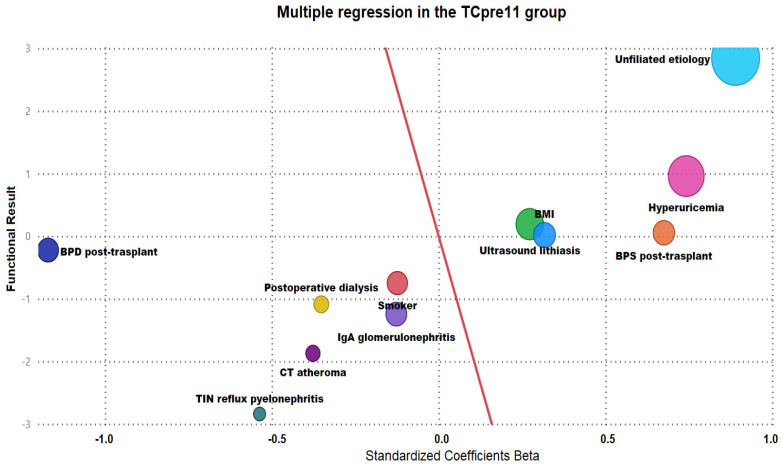
Multiple regression analysis between variables and kidney graft functional outcomes in the deceased donor transplant group prior to implementation of the living donor program.

**Figure 4:**
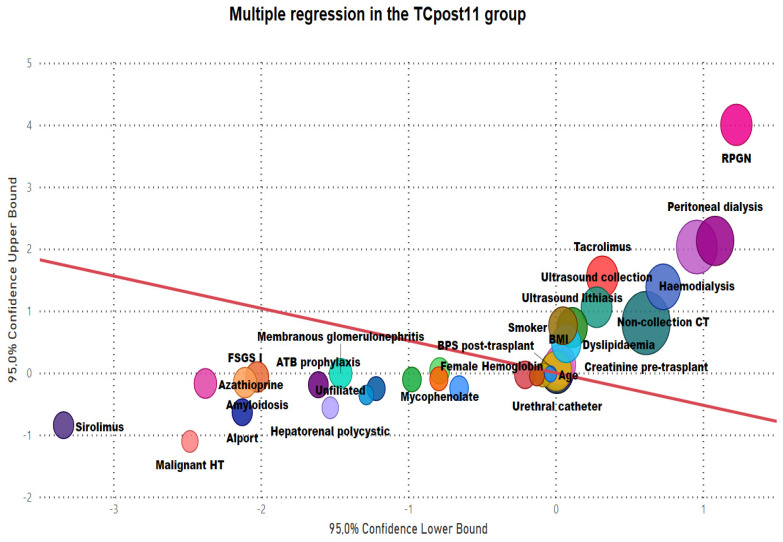
Multiple regression analysis between variables and kidney graft functional outcomes in the deceased donor transplant group after implementation of the living donor program.

Multivariate multiple regression analysis was performed on the general sample and each of the groups. Figure 3 and Figure 4 show the results for groups TCpre11 and TCpost11. In TCpost11, higher body mass index, tobacco smoking, not preventing lithiasis in the graft, allowing the progression of chronic renal failure with consequent increases in pre-transplant creatinine levels, insufficient control of pretransplant systolic blood pressure, less use of nonantibiotic prophylaxis for prevention of recurrent urinary tract infections, and increased time to removal of urethral catheter due to blockages were found to favour the deterioration of kidney function in the deceased donor program.

The LD program had a strong influence on deceased donor transplants in the prelysis phase.

The implementation of an LD program reduced the probability of acute rejection in TCpost11 patients. Similarly, the tendency towards normal renal function increased.

## 4. Discussion

Mean age was similar among the three groups in our study and approximately equal to the ages recorded in the national ONT registries (47.3 years for living donors compared to 46.6 years in the present study and 55.5 years for deceased donor transplant in the national registry compared to 56.6 in our sample). In a study conducted about kidney and pancreas transplant, Regmi et al. presented a trend towards accepting recipients who are older (up to 80 years of age in our sample), more obese, and more frail [10]. With regards to sex, 62.08% of recipients were male compared to 37.92% female recipients, while in published data, 59% of recipients were male [11]; these numbers become more extreme if we consider living donor transplants, where 69.33% of graft recipients were men and 30.67% were women, in contrast to published data where up to 59% of living donor recipients were women [12].

Veroux et al. acknowledge that BMI is a risk factor in transplant outcomes [13], and in this study, BMI was higher in TCpre11 (26.04) than in TCpost11 (25.67) and TV (24.17), which contrasts with numerous publications which describe a doubling in the number of obese patients at the end of the last century [14], although this discrepancy may be due to the differing origins of the samples, since most publications refer to American or Central and North European populations as opposed to the primarily Mediterranean population of the present study. This increase in BMI is key, as there is a significant relationship between obesity and the risk of graft loss and patient death [14]. The profile of kidney transplant candidates was, according to the present series, more favourable during the period before the living donor program was implemented, with the current profile featuring patients with greater body weight, which is associated with more comorbidities, including comorbidities caused by excess weight (more cases of acute rejection, delayed graft function, loss of graft function, and diabetes). Yin, S. et al. encountered this relationship between clinical outcomes after kidney transplantation [15] and comorbidities derived from the proinflammatory state induced by obesity. 

Patients in TCpre11 had more pretransplant infections. After the living donor program was implemented, more consideration has been given to prophylaxis and treatment of pretransplant UTIs, which comes hand in hand with the results obtained from the study of pretransplant UTIs, which are more frequent in TCpre11 than in either TV or TCpost11. Further research should be carried out in this area, as patients who are kidney transplant candidates or have undergone a transplant are a vulnerable group for whom infections have more severe repercussions, including kidney failure and death. It would therefore be advisable to prevent infections via measures such as vaccines or nonantibiotic prophylaxis [16].

In a systematic review, recipients with urinary tract infections exhibited the most frequent complications following a kidney transplant [17]. Antibiotic therapy, sublingual vaccines, and biopharmaceuticals such as oral mannose and its derivatives were used more in TCpre11 than in TV. No treatment or measure was used in a greater proportion in TV than in TCpre11 and TCpost11, which appears reasonable, as fewer UTIs were found in TV. Since the living donor program was implemented, more attention has been given to pretransplant UTIs, and preventing these conditions is associated with the use of products such as oral mannose and its derivatives [16] for increased protection of the kidney function of the graft. Factors such as the type of pretransplant dialysis (haemodialysis versus peritoneal dialysis) have not been shown to promote further infections [18]. Post-transplant creatinine (given one year after the transplant), a predictor of long-term transplant outcomes [19], was higher in TCpost11. Curiously, post-transplant creatinine is higher in TV (1.40) and TCpost11 (1.60) than in TCpre11 (1.30).

Hicks, M. et al. suggest that success in terms of transplanted organ function depends on the quality of the organ itself [20], successful organ function may depend on a number of factors, such as donor age, previous diseases and treatments, the mechanism of death and donor management in the hours leading up to the donation, cold ischemia time, and the circumstances of reperfusion [20]. It is precisely for this reason that creatinine levels in TCpost11 patients might be attributed to three cases which featured poor ischemia times and functional outcomes, and which were not excluded by the system during the statistical analysis.

In a meta-analysis conducted by Ming, Y. et al., it was concluded that patients who received a graft from a donor after brain death showed earlier graft function than those with grafts from a donor after controlled cardiac death, where warm ischemia time is longer [21]; in spite of this initial advantage, the medium-to-long term outcomes are comparable [21]. Mean ischemia time was 17.97 h, with a range of 6–29 h, and in line with data from large samples published in the literature [22] and was longer in TCpost11. With regards to cold ischemia time, some studies deny any impact on the recipient if cold ischemia time is less than 16 h [23], while Gorayeb-Polacchini, FS et al. found no significant differences between patients whose grafts had a cold ischemia time over versus under 20 h [24], and still, other series report that cold ischemia time above 36 h is not associated with worse outcomes in terms of kidney function [25]. In a retrospective study with outcomes of kidney transplant from deceased donors with acute kidney injury and prolonged cold ischemia time, an increased delay in kidney function if those 36 h are exceeded [26] is described, with a consequent increase in hospitalisation time [27], although Vinson, AJ. et al. pointed out that cold ischemia of over 20 h could also play a beneficial role in HLA matching [28]. 

Since living donor transplants were implemented, longer ischemia times have been adopted, with no statistical difference in outcomes between TCpre11 and Tcpost11. These outcomes are affected by various factors, such as the condition of the recipient, the use of bench surgery, an increase in organ transport times due to the transport of organs within the voluntary donor network, and the donor’s geographical location, which delay graft function [29].

The more frequent causes of graft failure were impaired kidney function in TV and graft complications that do not involve loss in groups TCpre and Tcpost; good functional results were more frequent in the TV group, compared with another study where Dolores Redondo. et al. targeted the evolution of the causes of graft loss in kidney transplant over 40 years where the most frequent cause was loss of kidney function with return to dialysis or retransplantation [30]. In the multivariate analysis of TCpost11, longer ischemia time was not observed to be related to deteriorating kidney function.

Many authors point out that another possible factor which may influence this aspect is the increase in postsurgical urethral catheterisation time since 2011, to 12.64 days with a median of 8 days, in contrast with the 6 days of postsurgical urethral catheterisation in some series [30], although urine cultures were positive in up to 34.2% of cases from this date until the removal of the double-J stent [30]. Since the implementation of living donor transplants, there has been a tendency towards delaying removal of the urethral catheter and double-J stent, from 54 days in TCpre11 to 61 days in TCpost11, a very large delay when compared to series from the most recent meta-analyses, which describe “early catheter removal” as seven days or less and “late removal” as more than fifteen days [31], while more ambitious series have early removal at 5 days [32]. Double-J ureteral stents reduce the rate of complications derived from ureteral reimplantation [33]. 

A meta-analysis that studied early and late ureteric stent removal after kidney transplantation concluded that increased time until removal of the double-J ureteral stent from grafts in our study protected against deterioration of kidney function, and its use did not increase associated morbidity [34]. In spite of all this, Luján S. et al. raised the concern that, with respect to the systematic catheterization used in kidney transplant and transplant centres, a consensus has not been reached about the time needed before the removal of double-J ureteral stents [35]. This is supported by the perfusion results measured in the postoperative renal Doppler ultrasound, which are better in TCpost11 than in TCpre11. Good perfusion detected via post-transplant Doppler ultrasound was associated with prevention of kidney function deterioration.

The greatest impact of the living donor program was in having deceased donor transplants be performed during the predialysis period, i.e., in obtaining candidate transplant patients who had not begun or had spent as little time as possible in renal replacement therapy. The implementation of a living donor program decreased the likelihood of acute rejection in TCpos11 patients. Similarly, it provided an increase in tendency towards normal kidney function, i.e., better graft function in the short and long term. 

In a study carried out by the Nephrology Service, Parc de Salut Mar, Barcelona, Spain in 2020, it was concluded that recipients with kidney grafts from donors with expanded criteria had a higher rate of kidney graft failure, no primary function of the graft, and worse kidney function when compared to donors with standard criteria. However, patient and graft survival at 5 years is similar when compared to donors with standard criteria. Regarding the evolution of these kidney grafts with respect to acute kidney failure, there are no tools that help us predict it, including preimplantation biopsy [36]; in the centres where the study has been carried out, it is not part of the protocol to perform pretransplant biopsy in all donors, only those who have some criterion that may affect the kidney function of the graft (high blood pressure, diabetes mellitus, etc.). The authors find this topic a limitation of this study, and this could be expanded upon in a substudy with collection of histological data and a comparison between a group of donors with expanded criteria and donors with standard criteria.

## 5. Conclusions

The implementation of a living donor transplant program influences the functional outcomes of deceased donor transplants.

The implementation of a living donor transplant program reduces the likelihood of acute rejection in the deceased donor transplant patient. In addition, it increases the tendency towards normal renal function in the kidney transplant recipient.

The probability of deterioration in kidney graft function is reduced with nonantibiotic prophylaxis for recurrent urinary tract infections, smoking cessation, increasing the number of days the double-J ureteral stent is left in the graft, and performing the transplant as soon as possible after the onset of kidney failure, preferably pre dialysis. 

## Data Availability

The raw data supporting the conclusions of this article will be made available by the authors on request.

## Supplementary Materials

The following supporting information can be downloaded at: https://www.mdpi.com/article/10.3390/jpm14040408/s1.
