# Supplementary material for: Enhancing Kidney Transplant Outcomes: The Impact of Living Donor Programs
Source: J Pers Med. 2024 Apr 12;14(4):408. doi: 10.3390/jpm14040408 (PMC11051259; doi:10.3390/jpm14040408)
Supplement: Supplementary file 1 [file jpm-14-00408-s001.zip › jpm-2933351-supplementary.pdf]

Supplementary Tables

Table S1.- Age, BMI, pre-transplant haemoglobin, pre-transplant creatinine, urine culture, post-transplant haemoglobin, post-transplant creatinine, pre-transplant systolic blood pressure, pre-transplant diastolic blood pressure, post-transplant systolic blood pressure, post-transplant diastolic blood pressure, ischemia time, duration of urethral catheterisation (days), duration of double-J stent (days)

Variable	Group	Mean	SD	Median	Range	p-value
**Age**	**TV**	46.62	14.69	48.00	18.00-74.00	0.0004
	**TCpre11**	57.30	16.24	60.00	21.00-80.00	
	**TCpost11**	56.91	13.75	59.00	18.00-80.00	
**BMI**	**TV**	24.92	3.59	24.17	18.22-38.06	0.0002
	**TCpre11**	27.03	4.97	26.04	19.43-38.09	
	**TCpost11**	26.81	4.75	25.67	17.50-42.78	
**Pre-transplant haemoglobin**	**TV**	12.05	1.89	11.60	9.00-17.80	0.060
	**TCpre11**	12.00	1.47	12.20	8.00-14.50	
	**TCpost11**	12.37	2.37	12.40	7.00-17.80	
**Pre-transplant creatinine**	**TV**	7.00	8.64	5.83	0.60-61.60	0.007
	**TCpre11**	2.27	1.62	1.60	0.70-6.80	
	**TCpost11**	1.98	1.50	1.50	0.63-9.70	
**Urine culture**	TV	1.00	0.69	1.00	0-5	0.0047
	TCpre11	3.67	3.33	3.00	0-13	
	TCpost11	3.01	3.25	1.00	0-13	
**Post-transplant haemoglobin**	**TV**	12.10	1.78	12.20	11.56-15.80	0.056
	**TCpre11**	12.05	1.27	12.10	12.00-15.50	
	**TCpost11**	12.30	1.37	12.34	11.20-16.80	
**Post-transplant creatinine**	**TV**	1.41		1.26	1.41	0.0026
	**TCpre11**	1.30	0.52	1.30	0.80-2.70	

	**TCpost11**	1.60	1.25	1.60	0.70-9.70	
**Pre-transplant systolic blood pressure**	**TV**	134.07	15.40	130.00	101-185	0.0001
	**TCpre11**	133.39	20.05	130.00	99-163	
	**TCpost11**	138.80	18.17	137.00	73-190	
**Pre-transplant diastolic blood pressure**	**TV**	82.32	9.52	80	60-110	0.0074
	**TCpre11**	73.99	12.64	72	48-98	
	**TCpost11**	78.42	12.22	80	49-111	
**Post-transplant systolic blood pressure**	**TV**	124.69	6.95	122.00	101-140	0.0001
	**TCpre11**	124.99	10.72	130.00	100-140	
	**TCpost11**	127.72	9.23	130.00	100-145	
**Post-transplant diastolic blood pressure**	**TV**	81.02	7.51	80.00	60-97	0.0002
	**TCpre11**	74.17	10.51	72.00	60-96	
	**TCpost11**	76.90	9.05	80.00	60-95	
**Ischemia time**	**TV**	10.05	2.63	10.00	6-14	0.0001
	**TCpre11**	18.73	5.45	20.00	7-29	
	**TCpost11**	19.36	3.68	18.00	13-28	
**Urethral catheter duration (days)**	**TV**	7.95	1.98	7.00	6-12	0.0003
	**TCpre11**	8.66	3.79	8.00	6-30	
	**TCpost11**	19.06	22.48	10.00	6-98	

**Double-J stent duration (days)**	**TV**	58.33	16.11	60.00	30-85	0.0006
	**TCpre11**	50.64	12.86	54.00	30-80	
	**TCpost11**	62.21	23.34	61.00	24-180	

Table S2. SEX. PRE-TRANSPLANT UTI. UTI TREATMENT. CONCOMITANT DISEASES. SMOKER. TYPE OF DIALYSIS. ACUTE REJECTION. FUNCTIONAL OUTCOME.

**GROUPS**	TV and TCpre11							TV and TCpost11							TCpre11 and TCpost11						
	n	%	n	%	n	%	p-value	n	%	N	%	n	%	p-value	n	%	n	%	n	%	p-value
**SEX**	**TV, n=150**		**TCpre11, n=650**		**Total, n=800**			**TV, n=150**		**TCpost11, n=500**		**Overall, n=650**			TC pre11, n=650		TC post11, n=500		Total, n=1150		
**Female**	46	30.67	267	41.08	313	39.13	0.0203	46	30.67	180	36	226	34.77	0.2421	267	41.08	180	36	447	38.87	0.0876
**Male**	104	69.33	383	58.92	487	60.88	0.0203	104	69.33	320	64	424	65.23	0.2421	383	58.92	320	64	703	61.13	0.0876
**PRE-TRANSPLANT UTI**	**TV, n=150**		**TCpre11, n=650**		**Total, n=800**			**TV, n=150**		**TCpost11, n=500**		**Total, n=650**			TC pre11, n=650		TC post11, n=500		Total, n=1150		
**Pre-transplant UTI**	18	12	167	25.69	185	23.13	0.0002	18	12.00	46	9.2	64	9.85	0.3481	167	25.69	46	9.2	213	18.52	
**UTI TREATMENT**	**TV, n=150**		**TCpre11, n=650**		**Total, n=800**			**TV, n=150**		**TCpost11, n=500**		**Total, n=650**			TC pre11, n=650		TC post11, n=500		Total, n=1150		
**UTI treatment**	n	%	n	%	n	%	p-value	n	%	n	%	n	%	p-value	n	%	n	%	n	%	p-value
**Antibiotic**	2	1.33	100	15.38	102	12.75	0.0108	2	1.33	24	4.8	26	4	0.0593	100	66.46	24	4.8	124	10.78	0.0001

**prophylaxis**																					
**Antibiotic demand**	16	10.67	432	66.46	448	56	0.0001	16	10.67	149	29.8	165	25.38	0.0001	432	5.08	149	29.8	581	50.52	0.0001
**Vaccine**															33	5.08	11	2.2	44	3.83	0.0127
**Mannose**															33	5.08	11	2.2	44	3.83	0.0127
**None**	132	88	118	18.15	250	31.25	0.0001	132	88.00	323	64.6	455	70	0.0001	118	18.15	323	64.6	441	38.35	0.0001
**CONCOMITANT DISEASES**	**TV, n=150**		**TCpre11, n= 650**		**Total, n=800**			**TV, n=150**		**TCpost11, n= 500**		**Total, n=650**			TC pre 11, n= 650		TC post11, n= 500		Total, n=1150		
**HT**	124	82.67	551	84.77	675	84.38	0.5334	124	82.67	444	88.8	568	87.38	0.0507	551	84.77	444	88.8	995	86.52	0.0551
**DM2**	27	18	133	20.46	160	20	0.5715	27	18.00	173	34.6	200	30.77	0.0001	133	20.46	173	34.6	306	26.61	0.0001
**Dyslipidemia**	79	52.67	316	48.62	395	49.38	0.4150	79	52.67	280	56	359	55.23	0.5125	316	48.62	280	56	596	51.83	0.0146
**Hyperuricemia**	45	30	183	28.15	228	28.5	0.6883	45	30.00	131	26.2	176	27.08	0.4020	183	28.15	131	26.2	314	27.3	0.5044
**Former smoker**	10	6.67	83	12.77	93	11.63	0.0344	10	6.67	102	20.4	112	17.23	0.0001	83	12.77	102	20.4	185	16.09	0.0006
**Smoker**	10	6.67	68	10.46	78	9.75	0.1723	10	6.67	56	11.2	66	10.15	0.1237	68	10.46	56	11.2	124	10.78	0.7021
**TYPE OF PREOPERATIVE DIALYSIS**	**TV, n=150**		**TCpre11, n=650**		**Total, n=800**			**TV, n=150**		**TCpost11, n=500**		**Total, n=650**			TC pre 11, n=650		TC post11, n=500		Total, n=1150		
**Peritoneal dialysis**	6	4	84	12.92	90	11.25	0.0009	6	4.00	79	15.8	85	13.08	0.0001	84	12.92	79	15.8	163	14.17	0.1731
**Haemodialysis**	43	28.67	465	71.54	508	63.5	0.0001	43	28.67	172	34.4	215	33.08	0.1998	465	71.54	172	34.4	637	55.39	0.0001
**None**	101	67.33	134	20.62	235	29.38	0.0001	101	67.33	297	59.4	398	61.23	0.0859	134	20.62	297	59.4	431	37.48	0.0001
**POSTOPERATIVE**	**TV, n=150**		**TCpre11, n=650**		**Total, n=800**		p-value	**TV, n=150**		**TCpost11,**		**Total, n=650**		p-value	**TC pre 11,**		**TC post11,**		**Total, n=1300**		p-value

DIALYSIS									n=500					n=650		n=500				
**Yes**	49	32.67	167	25.69	216	27	0.0840	49	32.67	109	21.8	158	24.31	0.0090	167	25.69	109	21.8	276	24	0.1435
**No**	101	67.33	483	74.31	584	73	0.0840	101	67.33	391	78.2	492	75.69	0.0090	483	74.31	391	78.2	874	76	0.1435
**ACUTE REJECTION**	**TV, n=150**		**TCpre11, n=650**		**Total, n=1300**			**TV, n=150**		**TCpost11, n=500**		**Total, n=1300**			**TC pre 11, n=650**		**TC post11, n=500**		**Total, n=1300**		
**Yes**	14	9.33	96	14.77	110	13.75	0.0330	14	9.33	49	9.8	63	9.69	0.6318	96	14.77	49	9.8	145	12.6086957	0.0121
**No**	136	90.67	554	85.23	690	86.25	0.0330	136	90.67	451	90.2	587	90.31	0.6318	554	85.23	451	90.2	1005	87.3913043	0.0121
**FUNCTIONAL OUTCOME**	**TCpre11, n=650**		**TCpost11, n=500**		**Total, n=1300**			**TV, n=150**		**TCpost11, n=500**		**Total, n=1300**			**TV, n=150**		**TC pre 11, n=650**		**Total, n=1300**		
**Good**	234	36.00	332	66.4	566	49.2173913	0.0001	133	88.67	332	66.4	465	71.54	0.0001	133	88.67	234	36.00	367	45,875	0.0001
**Grafting complications****	332	51.08	104	20.8	436	37.9130435	0.0001	1	0.67	104	20.8	105	16.15	0.0001	1	0.67	332	51.08	333	41,625	0.0001
**Impaired kidney function**	67	10.31	20	4	87	7.56521739	0.0001	11	7.33	20	4	31	4.77	0.1232	11	7.33	67	10.31	78	9.75	0.3587
**Graft loss**	17	2.62	44	8.8	61	5.30434783	0.0001	3	2.00	44	8.8	47	7.23	1.0000	3	2.00	17	2.62	20	2.5	1.0000
